# Propensity-matched analysis of three different chemotherapy sequences in patients with locoregionally advanced nasopharyngeal carcinoma treated using intensity-modulated radiotherapy

**DOI:** 10.1186/s12885-015-1768-x

**Published:** 2015-10-27

**Authors:** Wen-Fei Li, Ying-Qin Li, Lei Chen, Yuan Zhang, Rui Guo, Fan Zhang, Hao Peng, Ying Sun, Jun Ma

**Affiliations:** Department of Radiation Oncology, Sun Yat-sen University Cancer Center, State Key Laboratory of Oncology in South China, Collaborative Innovation Center for Cancer Medicine, 651 Dongfeng Road East, Guangzhou, 510060 People’s Republic of China

**Keywords:** Nasopharyngeal carcinoma, Concurrent chemoradiotherapy, Induction chemotherapy, Intensity-modulated radiotherapy

## Abstract

**Background:**

To compare the survival outcomes and acute toxicities of concurrent chemoradiotherapy (CCRT), induction chemotherapy (IC) plus radiotherapy (RT), and IC plus CCRT in patients with locoregionally advanced nasopharyngeal carcinoma (NPC) treated using intensity-modulated radiotherapy (IMRT).

**Methods:**

Patients with stage III–IVB NPC who were treated with IMRT between 2009 and 2012 at a single institution were retrospectively reviewed. The induction regimens included PF (cisplatin and fluorouracil) and TP (docetaxel and cisplatin) every 3 weeks for 2–3 cycles; the concurrent regimen was cisplatin every three weeks for 2–3 cycles. A propensity score matching method was used to match patients from each group in a 1:1:1 ratio.

**Results:**

In total, 147 eligible patients were propensity-matched, with 49 patients in each treatment group. The median follow-up duration was 38.5 months (range, 4.5 – 56 months). The 3-year disease-free survival, overall survival, distant metastasis-free survival, and locoregional relapse-free survival rates were 82.1 %, 92.8 %, 87 %, and 90.4 % in the CCRT group; 86.3 %, 91.0 %, 91.6 %, and 94.4 % in the IC plus RT group; and 87.8 %, 95.8 %, 93.8 %, and 93.9 % in the IC plus CCRT group, respectively. No statistically significant survival differences were observed between the three treatment groups in either univariate or multivariate analyses. The incidence of grade 3–4 acute toxicities was similar among groups.

**Conclusions:**

This study suggests that CCRT, IC plus RT, and IC plus CCRT are similarly efficacious treatment strategies for patients with locoregionally advanced NPC treated using IMRT; however, long-term, large-scale randomized trials are required to confirm these findings.

## Background

Nasopharyngeal carcinoma (NPC) is the most common malignant head and neck cancer in Southern China, and over 70 % of cases of newly-diagnosed NPC are classified as locoregionally advanced disease [1, 2]. Concurrent chemoradiotherapy (CCRT) is the standard treatment for locoregionally advanced NPC. Randomized trials [3–9] and meta-analyses [10–12] have demonstrated that CCRT can significantly improve locoregional and distant control compared to radiotherapy (RT) alone, which has ultimately improved overall survival (OS) in locoregionally advanced NPC.

Induction chemotherapy (IC) before RT may also reduce the risk of locoregional recurrence and distant metastasis in NPC [13]. Compared to concurrent chemotherapy, IC may offer the benefits of early eradication of micrometastases and reduction of the tumor burden, and not increase toxicities during RT. Although the results of randomized trials investigating the value of adding IC to RT [14–17] or CCRT [18–20] are controversial, a meta-analysis of these trials indicated that IC could effectively reduce the rate of distant metastasis and improve OS in locoregionally advanced NPC [21].

Intensity-modulated radiotherapy (IMRT) has now replaced two-dimensional conventional radiotherapy (2DCRT) as the mainstay RT technique for NPC. Compared to 2DCRT, IMRT leads to significantly better treatment outcomes by achieving a higher local control rate. However, distant metastasis has become the major treatment failure pattern in NPC [22–24]. Although IC and concurrent chemotherapy may both improve this, a direct comparison of these two approaches has not been conducted in patients with NPC treated using IMRT. Moreover, it remains uncertain whether combining IC and CCRT can further reduce the risk of distant metastasis. Therefore, we conducted a retrospective study to compare the survival outcomes and acute toxicities of CCRT, IC plus RT, and IC plus CCRT in patients treated with IMRT, in order to help guide treatment strategy selection for patients with locoregionally advanced NPC.

## Methods

### Patients

Patients with newly diagnosed, non-distant metastatic, histologically-proven NPC treated with IMRT at our Cancer Center between October 2009 and February 2012 were retrospectively reviewed. The pre-treatment evaluation included a complete patient history, physical examination, hematology and biochemistry profiles, nasopharyngeal fiberoptic endoscopy, MRI of the nasopharynx and neck, chest radiography, abdominal sonography and whole body bone scan using 99mTc-methyldiphosphonate single photon emission computed tomography (SPECT). Only patients with stage III-IVB disease according to the 7^th^ edition of the UICC/AJCC staging system [25] who received the study-defined IC or CCRT regimens were included in this study. The patients were classified into three treatment groups: (1) CCRT group, (2) IC plus RT group, and (3) IC plus CCRT group. The study was approved by the Institutional Review Board of Sun Yat-sen University Cancer Center. All patients provided written informed consent for participation in the study and analysis of their medical records.

### Radiotherapy

All patients received radical IMRT to treat the nasopharyngeal and neck tumor volumes for the entire treatment course. All patients were immobilized in the supine position using a head, neck and shoulder thermoplastic mask. Intravenous contrast-enhanced CT simulation was performed at 3 mm intervals from the head to 2 cm below the sternoclavicular joint using a CT simulator. Target volumes were delineated slice-by-slice on treatment planning CT scans according to an individualized delineation protocol, in accordance with the International Commission on Radiation Units and Measurements reports 50 and 62. The prescribed doses were 68–72 Gy in 30–33 fractions to the planning target volume (PTV) of the primary gross tumor volume (GTVnx), 64–70 Gy to the PTV of the GTV of the involved lymph nodes (GTVnd), 60–63 Gy to the PTV of the high-risk clinical target volume (CTV1), and 54–56 Gy to the PTV of the low-risk clinical target volume (CTV2). All targets were treated simultaneously using the simultaneous integrated boost technique.

### Chemotherapy

During the study period, the institutional guidelines recommended CCRT ± induction and/or adjuvant chemotherapy for stage III to IVB disease. Reasons for deviating from the institutional guidelines included organ dysfunction suggesting intolerance to chemotherapy, patient refusal, and the discretion of the doctors in individual cases. The study-defined IC regimens included PF (80 mg/m^2^ cisplatin on day 1 and 800 mg/m^2^/d fluorouracil civ on days 1–5) or TP (75 mg/m^2^ docetaxel on day 1 and 75 mg/m^2^ cisplatin on day 1); both regimens were repeated every 3 weeks for 2–3 cycles. The study-defined CCRT regimen was 80–100 mg/m^2^ cisplatin on day 1 every 3 weeks for 2–3 cycles. Patients receiving other chemotherapy regimens or who received only one cycle of induction or concurrent chemotherapy were excluded from this study.

### Follow-up and statistical analysis

Patient follow-up was measured from the first day of therapy to the day of last examination or death. Patients were examined at least every 3 months during the first 2 years, with follow-up examinations every 6 months for 3 years or until death. Disease–free survival (DFS) was calculated from day 1 of treatment to locoregional relapse, distant relapse or tumor-related death, whichever occurred first. OS was calculated from day 1 of treatment to last examination or death; distant metastasis–free survival (DMFS) and locoregional relapse–free survival (LRRFS), to first distant metastasis and locoregional relapse, respectively.

All statistical analyses were performed using SPSS v 18.0 (Chicago, IL, USA). A propensity score matching method [26, 27] was used to match the patients from each of the three groups (CCRT, IC plus RT, and IC plus CCRT) in a 1:1:1 ratio. Categorical variables were compared using the Chi–square test (or Fisher’s exact test, if the expected number was less than five in at least 25 % of the cells), and continuous variables were compared using ANOVA. Survival rates were calculated using the Kaplan-Meier method and the log-rank test was used to perform paired comparisons between each of the treatment groups using the pair-wise over strata method. Multivariate analyses with the Cox proportional hazards model [28] were used to calculate hazard ratios (HR), 95 % confidence intervals (CI) and test the independent significance of different factors by backward elimination of insignificant variables. Two-tailed *P*-values < 0.05 were considered statistically significant; *P* -value corrections for multiple comparisons were not performed.

## Results

### Characteristics of the patients in the propensity-matched groups

A total of 305 eligible patients were enrolled. CCRT was delivered to 198 patients, IC plus RT to 49 patients, and IC plus CCRT to 58 patients. In the 49 patients in the IC plus RT group, the reasons for not receiving concurrent chemotherapy were as follows: bone marrow depression (8/49, 16.3 %), liver dysfunction (3/49, 6.1 %), patient refusal (21/49, 42.9 %), and doctors’ discretion (17/49, 34.7 %). The characteristics of the patients were not balanced in the three treatment groups: the percentage of patients with stage IVA-B disease was higher in the IC plus CCRT group (27/58, 46.6 %) than in the IC plus RT group (18/49, 36.7 %) and then in the CCRT group (46/198, 23.2 %, *P* = 0.002). Clinical variables including sex, age, T-category, N-category and overall stage were used to generate a propensity score model. Eventually, 147 patients were propensity matched to create three groups each containing 49 patients. The characteristics of the patients were well-balanced between the propensity-matched groups (Table 1).

**Table 1:** Characteristics of the 147 propensity-matched patients

Characteristic	CCRT group (*n =* 49)	IC plus RT group (*n* = 49)	IC plus CCRT group (*n* = 49)	*P value* ^*^
Sex				0.672
Male	32 (65.3 %)	31 (63.3 %)	35 (71.4 %)	
Female	17 (34.7 %)	18 (36.7 %)	14 (28.6 %)	
Age (years)				0.696
≤45	19 (38.8 %)	20 (40.8 %)	23 (46.9 %)	
>45	30 (61.2 %)	29 (59.2 %)	26 (53.1 %)	
Histological type			-	
WHO type I	0 (0 %)	0 (0 %)	0 (0 %)	
WHO type II/III	49 (100 %)	49 (100 %)	49 (100 %)	
T-category				1.000
T1	1 (2 %)	0 (0 %)	0 (0 %)	
T2	1 (2 %)	2 (4.1 %)	2 (4.1 %)	
T3	32 (65.3 %)	33 (67.3 %)	32 (65.3 %)	
T4	15 (30.6 %)	14 (28.6 %)	15 (30.6 %)	
N-category				0.956
N0	4 (8.2 %)	4 (8.2 %)	4 (8.2 %)	
N1	29 (59.2 %)	27 (55.1 %)	28 (57.1 %)	
N2	10 (20.4 %)	13 (26.5 %)	9 (18.4 %)	
N3	6 (12.2 %)	5 (10.2 %)	8 (16.3 %)	
Stage-group				0.891
III	31 (63.3 %)	31 (63.3 %)	29 (59.2 %)	
IVA-B	18 (36.7 %)	18 (36.7 %)	20 (40.8 %)	

Abbreviations: *IC* induction chemotherapy, *RT* radiotherapy, *CCRT* concurrent chemoradiotherapy

^*^P-values were calculated using the Chi-square test (or Fisher’s exact test, if the expected number was less than five in at least 25 % of the cells)

All patients completed the planned IMRT protocol. The median RT dose was 68 Gy (range, 68–72) in the CCRT group, and 70 Gy (range, 68–72) in the other two groups. All patients received at least two cycles of chemotherapy; more patients in the IC plus RT group received three cycles of IC than patients in the IC plus CCRT group (32.7 % vs. 20.4 %), and more patients in the CCRT group received three cycles of concurrent cisplatin than patients in the IC plus CCRT group (18.4 % vs. 8.2 %). However, the differences were not statistically significant (Table 2). Of the 98 patients receiving IC, 57 (58.2 %) received the PF regimen and 41 (41.8 %) received the TP regimen; patient characteristics were similar between the PF and TP groups (data not shown).

**Table 2:** Summary of treatments for the 147 propensity-matched patients

Treatment	CCRT group (*n* = 49)	IC plus RT group (*n* = 49)	IC plus CCRT group (*n* = 49)	*P* value
RT dose (Gy)				0.004^*^
Median (range)	68 (68–72)	70 (68–72)	70 (68–72)	
RT days				0.419^*^
Median (range)	44 (40–54)	43 (40–54)	44 (40–55)	
IC regimen				0.838^†^
PF	-	28 (57.1 %)	29 (59.2 %)	
TP	-	21 (42.9 %)	20 (40.8 %)	
IC cycles				0.170^†^
Two cycles	-	33 (67.3 %)	39 (79.6 %)	
Three cycles	-	16 (32.7 %)	10 (20.4 %)	
CCRT cycles				0.233^†^
Two cycles	40 (81.6 %)	-	45 (91.8 %)	
Three cycles	9 (18.4 %)	-	4 (8.2 %)	

Abbreviations: *IC* induction chemotherapy, *RT* radiotherapy; *CCRT* concurrent chemoradiotherapy

^*^P-values were calculated using ANOVA

^†^*P*-values were calculated using the Chi-square test (or Fisher’s exact test, if the expected number was less than five in at least 25 % of the cells)

### Survival outcomes

**Fig. 1:**
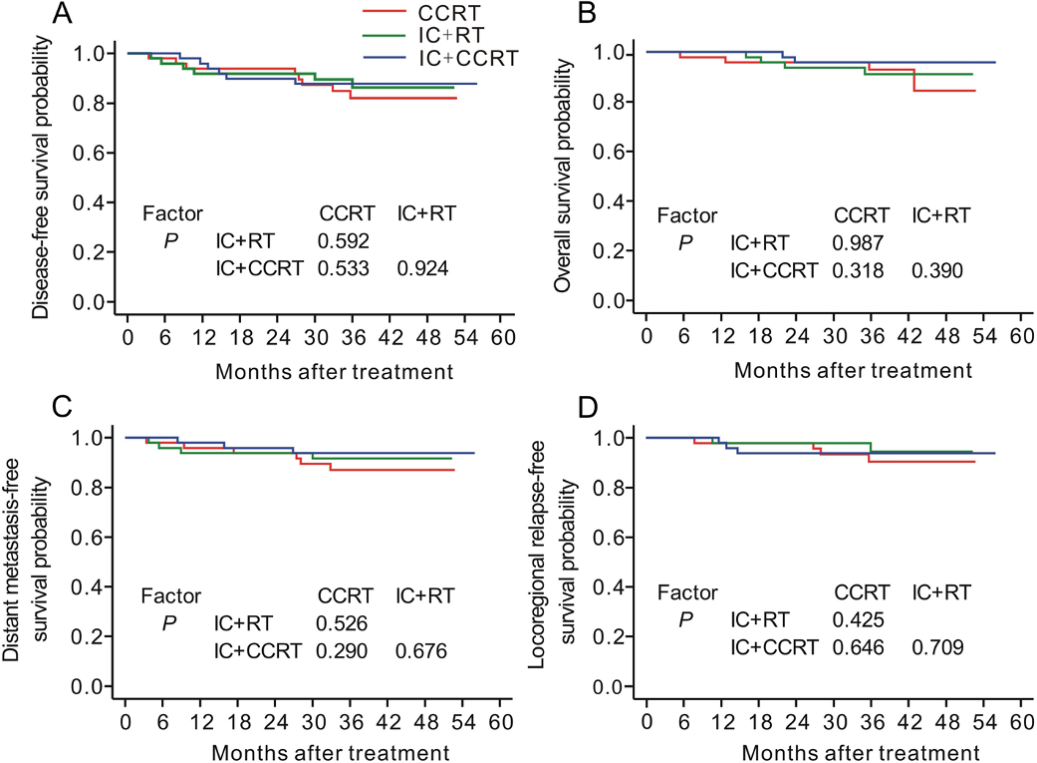
Kaplan–Meier survival curves. Disease–free survival (**a**), overall survival (**b**), distant metastasis–free survival (**c**) and locoregional relapse–free survival (**d**) for the CCRT group, IC plus RT group and IC plus CCRT group. *P*-values were calculated using the unadjusted log–rank test. IC, induction chemotherapy; RT, radiotherapy; CCRT, concurrent chemoradiotherapy

The median follow-up time for the 147 propensity score-matched patients was 38.5 months (range, 4.5–56 months). A total of 20/147 (13.6 %) patients experienced treatment failure or death, nine (6.1 %) experienced locoregional recurrence, 13 (8.8 %) experienced distant metastasis, and 10 (6.8 %) patients died. For the CCRT group, the 3-year DFS, OS, DMFS, and LRRFS rates were 82.1 %, 92.8 %, 87 %, and 90.4 %, respectively. For the IC plus RT group, the 3-year DFS, OS, DMFS, and LRRFS rates were 86.3 %, 91 %, 91.6 %, and 94.4 %, respectively. For the IC plus CCRT group, the 3-year DFS, OS, DMFS, and LRRFS rates were 87.8 %, 95.8 %, 93.8 %, and 93.9 %, respectively. No statistically significant survival differences were observed between the three treatment groups (Table 3, Fig. 1). Of the 98 patients receiving IC, those who received the TP regimen had similar 3-year DFS (p = 0.531), OS (*p* = 0.686), DMFS (*p* = 0.465) and LRRFS (*p* = 0.937) compared with patients who received the PF regimen.

**Table 3:** Comparison of the survival rates for each treatment group

Comparison	IC plus RT vs. CCRT	IC plus CCRT vs. CCRT	IC plus CCRT vs. IC plus RT
	(*n* = 98)	(*n =* 98)	(*n =* 98)
Three-year DFS	86.3 % vs. 82.1 %	87.8 % vs. 82.1 %	87.8 % vs. 86.3 %
*P* value^*^	0.592	0.533	0.924
Three-year OS	91 % vs. 92.8 %	95.8 % vs. 92.8 %	95.8 % vs. 91 %
*P* value^*^	0.987	0.318	0.390
Three-year DMFS	91.6 % vs. 87 %	93.8 % vs. 87 %	93.8 % vs. 91.6 %
*P* value^*^	0.526	0.290	0.676
Three-year LRRFS	94.4 % vs. 90.4 %	93.9 % vs. 90.4 %	93.9 % vs. 94.4 %
*P* value^*^	0.425	0.646	0.709

Abbreviations: *IC* induction chemotherapy, *RT* radiotherapy, *CCRT* concurrent chemoradiotherapy, *DFS* disease–free survival, *OS* overall survival, *DMFS* distant metastasis–free survival, *LRRFS*, Locoregional relapse–free survival

^*^P-values were calculated using the unadjusted log–rank test

### Multivariate analyses

Multivariate analyses were performed to further adjust for various prognostic factors. The following parameters were included in the Cox proportional hazards model: sex (female vs. male), age (>45 years vs. ≤45 years), T-category (T4 vs. T1-3), N-category (N2-3 vs. N0-1), RT dose (>68 Gy vs. 68 Gy) and chemotherapeutic intervention (IC plus CCRT vs. IC plus RT vs. CCRT). Multivariate analyses demonstrated that treatment group was not a significant prognostic factor for any endpoint (Table 4). N-category was an independent prognostic factor for DFS (*P* = 0.005) and DMFS (*P* = 0.001), and T-category was an independent prognostic factor for DFS (*P* = 0.048).

**Table 4:** Summary of multivariate analyses of prognostic factors in the 147 propensity-matched patients

Endpoint	Factor	HR	95 % CI	*P value* ^*^
Disease failure	Age >45 yrs vs. ≤45 yrs	1.30	0.48-3.53	0.603
	Sex female vs. male	0.59	0.21-1.65	0.310
	AJCC T-category T4 vs. T1-3	2.47	1.01-6.07	0.048
	AJCC N-category N2-3 vs. N0-1	3.72	1.49-9.25	0.005
	RT dose >68 Gy vs. 68 Gy	0.51	0.21-1.23	0.133
	Treatment group IC plus RT vs. CCRT	0.83	0.29-2.42	0.734
	Treatment group IC plus CCRT vs. CCRT	0.79	0.26-2.37	0.669
Death	Age >45 yrs vs. ≤45 yrs	2.69	0.57-12.76	0.212
	Sex female vs. male	1.46	0.40-5.35	0.572
	AJCC T-category T4 vs. T1-3	1.58	0.42-6.00	0.499
	AJCC N-category N2-3 vs. N0-1	2.97	0.84-10.54	0.092
	RT dose >68 Gy vs. 68 Gy	0.29	0.07-1.12	0.071
	Treatment group IC plus RT vs. CCRT	1.19	0.29-4.93	0.813
	Treatment group IC plus CCRT vs. CCRT	0.64	0.11-3.85	0.622
Distant failure	Age >45 yrs vs. ≤45 yrs	1.97	0.54-7.25	0.306
	Sex female vs. male	0.59	0.15-2.26	0.437
	AJCC T-category T4 vs. T1-3	2.97	0.98-9.00	0.054
	AJCC N-category N2-3 vs. N0-1	8.38	2.26-31.06	0.001
	RT dose >68 Gy vs. 68 Gy	0.53	0.18-1.57	0.248
	Treatment group IC plus RT vs. CCRT	0.67	0.19-2.41	0.541
	Treatment group IC plus CCRT vs. CCRT	0.48	0.11-2.00	0.310
Locoregional	Age >45 yrs vs. ≤45 yrs	0.62	0.15-2.52	0.506
failure	Sex female vs. male	0.59	0.12-2.86	0.514
	AJCC T-category T4 vs. T1-3	2.88	0.77-10.72	0.115
	AJCC N-category N2-3 vs. N0-1	1.31	0.32-5.41	0.705
	RT dose >68 Gy vs. 68 Gy	0.54	0.14-1.99	0.351
	Treatment group IC plus RT vs. CCRT	0.51	0.09-2.82	0.443
	Treatment group IC plus CCRT vs. CCRT	0.77	0.16-3.65	0.738

Abbreviations: *HR* hazard ratio, *CI* confidence interval, *IC* induction chemotherapy, *RT* radiotherapy, *CCRT* concurrent chemoradiotherapy

^*^P-values were calculated using the adjusted Cox proportional-hazards model

### Acute toxicities

No treatment-related deaths were observed in any group. Acute toxicities were similar between groups (Table 5); 32.7 % (16/49) of patients in the CCRT group, 38.8 % (19/49) of patients in the IC plus RT group, and 40.8 % (20/49) of patients in the IC plus CCRT group experienced grade 3–4 acute toxicities (*P* = 0.685). The most frequent grade 3 – 4 hematological toxicity was leucopenia in four patients (8.2 %) in the CCRT group, 10 patients (20.4 %) in the IC plus RT group, and 11 patients (22.4 %) in the IC plus CCRT group (*P* = 0.126). The most commonly recorded non-hematological adverse event was grade 3 – 4 mucositis in 11 patients (22.4 %) in the CCRT group, seven patients (14.3 %) in the IC plus RT group, and 12 patients (24.5 %) in the IC plus CCRT group (*P* = 0.415).

**Table 5:** Adverse events

Variable	CCRT group (*n =* 49)	IC plus RT group (*n =* 49)	IC plus CCRT group (*n =* 49)	*P* value^*^
Total Grade 3–4 acute adverse events	16 (32.7 %)	19 (38.8 %)	20 (40.8 %)	0.685
Hematologic				
Leukopenia	4 (8.2 %)	10 (20.4 %)	11 (22.4 %)	0.126
Neutropenia	3 (6.1 %)	9 (18.4 %)	9 (18.4 %)	0.135
Anemia	0 (0 %)	1 (2 %)	1 (2 %)	1.000
Thrombocytopenia	2 (4.1 %)	2 (4.1 %)	2 (4.1 %)	1.000
Non–hematologic				
Dermatitis	1 (2 %)	2 (4.1 %)	2 (4.1 %)	1.000
Mucositis	11 (22.4 %)	7 (14.3 %)	12 (24.5 %)	0.415
Dysphagia	1 (2 %)	0 (0 %)	1 (2 %)	1.000
Nausea/vomiting	2 (4.1 %)	2 (4.1 %)	3 (6.1 %)	1.000
Dry mouth	0 (0 %)	0 (0 %)	0 (0 %)	-
Ototoxicity	0 (0 %)	0 (0 %)	0 (0 %)	-
Hepatoxicity	0 (0 %)	3 (6.1 %)	1 (2 %)	0.324
Nephrotoxicity	0 (0 %)	0 (0 %)	0 (0 %)	-
Neurotoxicity	0 (0 %)	0 (0 %)	0 (0 %)	-

Abbreviations: *IC* induction chemotherapy, *RT* radiotherapy, *CCRT* concurrent chemoradiotherapy

^*^P-values were calculated using the Chi-square test (or Fisher’s exact test, if the expected number was less than five in at least 25 % of the cells)

## Discussion

To our knowledge, this is the first study to compare the survival outcomes and toxicities of CCRT, IC plus RT, and IC plus CCRT in patients with locoregionally advanced NPC treated using IMRT. All patients received at least two cycles of IC based on PF or TP regimens, and/or at least two cycles of concurrent chemotherapy based on cisplatin every three weeks. Moreover, a propensity score matching method was used to adjust for differences in the baseline characteristics of the patients and reduce selection bias, in order to enable an accurate comparison of the efficacies of these chemotherapy sequences. However, no statistically significant differences in DFS, OS, DMFS or LRRFS were observed between the three treatment groups.

Both induction and concurrent chemotherapy are effective treatment strategies for NPC [10–12, 21]; however, the optimal chemotherapy sequence that may further improve the survival rate in NPC remains to be identified. Only one phase 3 trial, by Xu et al., has compared IC plus RT and adjuvant chemotherapy versus CCRT plus adjuvant chemotherapy in locoregionally advanced NPC [29], in which 2DCRT was adopted as the RT technique and a PF regimen was used in the induction, concurrent and adjuvant phases. Both groups achieved similar outcomes. However, the combination of adjuvant chemotherapy may have narrowed the survival differences between the two treatment groups [29]. In two retrospective studies comparing IC plus RT with CCRT, no significant differences in survival were reported. However, the locoregional control rate seemed to be slightly better in the CCRT group than the IC plus RT group [30, 31]. However, none of these studies were entirely based on patients treated with IMRT.

All patients analyzed in this study received IMRT. The IC plus RT group had similar 3-year DFS (86.3 % vs. 82.1 %, *P =* 0.592), OS (91 % vs. 92.8 %, *P* = 0.987), DMFS (91.6 % vs. 87 %, *p* = 0.526), and LRRFS (94.4 % vs. 90.4 %, *P =* 0.425) rates compared to the CCRT group. These results suggest that IC plus RT is equivalent to CCRT in patients treated using IMRT, and that the improved locoregional control provided by IMRT may minimize the survival benefit of concurrent chemotherapy. However, it should be noted that higher-intensity regimens (PF or TP vs. cisplatin) were used and there were more patients who received three cycles of chemotherapy (32.7 % vs. 18.4 %) in the induction phase than in the concurrent phase; thus, the efficacy of IC versus concurrent chemotherapy in NPC patients treated with IMRT requires further evaluation.

IC plus CCRT has been proposed as a promising treatment strategy that may provide a survival benefit in locoregionally advanced NPC. The assumption is that increased cycles of chemotherapy could further reduce disease recurrence in high-risk patients. Several randomized trials have compared IC plus CCRT vs. CCRT [18–20] or IC plus CCRT vs. IC plus RT [32]. However, the efficacy of induction-concurrent strategies remains controversial. In a phase 2 study by Hui et al., addition of a TP-based IC regimen to CCRT significantly increased 3-year OS (94.1 % vs. 67.7 %, *P =* 0.012), and potentially improved progression–free survival and reduced distant metastasis compared to CCRT alone [18]. However, in two other randomized trials, IC using CEP (cisplatin, epirubicin, paclitaxel) [19] or GCP (gemcitabine, carboplatin, paclitaxel) [20] failed to show a survival benefit when added to CCRT. Huang et al. conducted a phase 3 randomized trial comparing IC plus CCRT with IC plus RT. No significant differences in survival were observed between the two treatment groups, and the authors concluded that concurrent carboplatin was the main reason for the negative results [32].

In this study, the IC plus CCRT group demonstrated no significant improvement in OS, DFS, DMFS or LRRFS over the CCRT group or IC plus RT group. Several factors could explain these negative results. Firstly, each treatment group had only 49 matched patients. The relatively small sample size may have meant the study was underpowered to detect differences in survival, especially with regards to NPC patients treated with IMRT. Secondly, only a few patients received three cycles of IC and concurrent 80–100 mg/m^2^ cisplatin in the IC plus CCRT group, which may have reduced the effectiveness of IC and CCRT [20]. Thirdly, truly high-risk patients who may benefit from more cycles of chemotherapy may be yet to be identified. Fourthly, it is possible that the use of other more effective chemotherapy regimens could provide additional survival benefit, such as induction TPF (docetaxel, cisplatin and fluorouracil) [33, 34] and PX (cisplatin and capecitabine) [35]. Therefore, the efficacy of IC plus CCRT requires further investigation.

The toxicity profiles of all three treatment groups were similar. There was a slightly lower incidence of grade 3–4 hematological toxicities in the CCRT group, and a lower incidence of grade 3–4 mucositis in the IC plus RT group; however, these differences were not significant. Notably, the incidence of grade 3–4 acute toxicities was lower than the rates reported in randomized trials [3–9, 18]; the main reasons for this observation may be the retrospective nature of this study, primary prophylaxis with granulocyte colony-stimulating factor, or inadequate monitoring of the adverse events in outpatients.

In this study, CCRT, IC plus RT, and IC plus CCRT led to similar survival outcomes and acute toxicities in patients with locoregionally advanced NPC treated using IMRT. However, it should be noted that this is a retrospective study with a relatively small sample size (*n* = 147) and a short follow-up (median, 38.5 months). Thus, the findings of this study require validation in phase 3 trials; the question of what chemotherapy should be given with IMRT for locoregionally advanced NPC remains unanswered.

## Conclusions

In conclusion, the results of this study suggest that CCRT, IC plus RT, and IC plus CCRT are similarly efficacious treatment strategies for patients with locoregionally advanced NPC treated using IMRT. The results of this study need to be confirmed by long-term, large-scale prospective trials.

## Abbreviations

NPC: Nasopharyngeal carcinoma
IMRT: Intensity-modulated radiotherapy
2DCRT: Two-dimensional conventional radiotherapy
RT: Radiotherapy
CCRT: Concurrent chemoradiotherapy
IC: Induction chemotherapy
OS: Overall survival
DFS: Disease–free survival
DMFS: Distant metastasis–free survival
LRRFS: Locoregional relapse–free survival
SPECT: Single photon emission computed tomography
PTV: Planning target volume
GTV: Gross tumor volume
CTV: Clinical target volume
HR: Hazard ratio
CI: Confidence interval
